# Supplementary material for: Comparison of Long-Read Methods for Sequencing and Assembly of Lepidopteran Pest Genomes
Source: Int J Mol Sci. 2022 Dec 30;24(1):649. doi: 10.3390/ijms24010649 (PMC9820851; doi:10.3390/ijms24010649)
Supplement: Supplementary file 1 [file ijms-24-00649-s001.zip › Supplementary Figures.pdf]

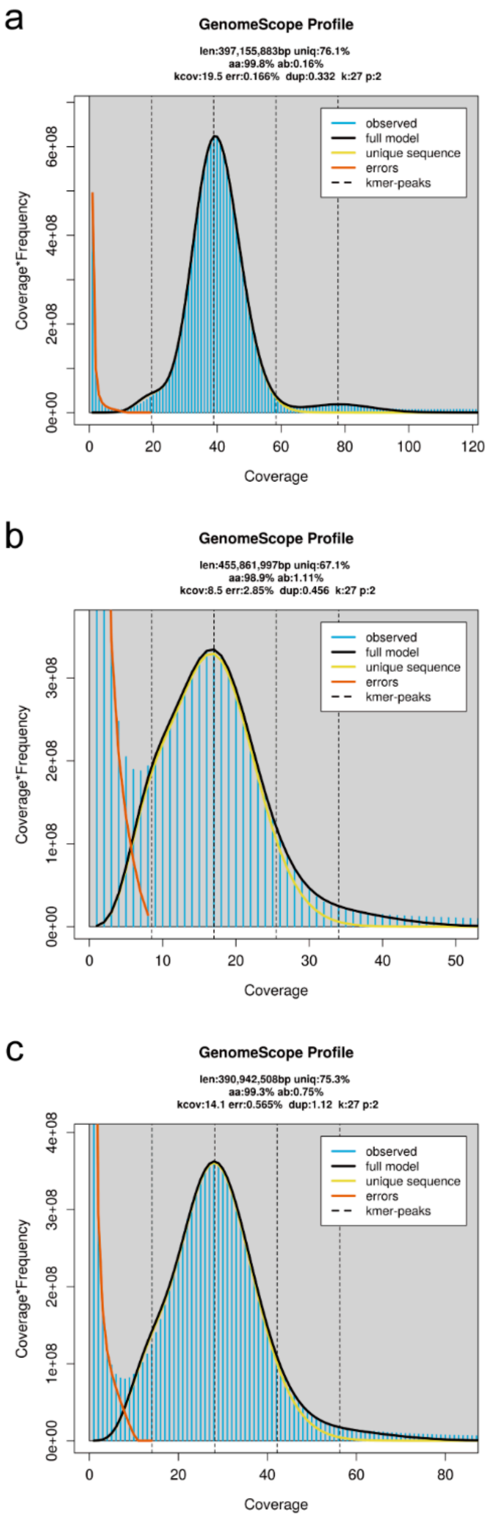

Supplementary Figure S1. GenomeScope plots for heterozygous species. （a）silkworm (D9L) PacBio Continuous Long Read (CLR) sequencing, (b) silkworm (D9L×N4) Oxford Nanopore Technologies（ONT）sequencing,（c）silkworm (P50T) PacBio High Fidelity (HIFI) sequencing

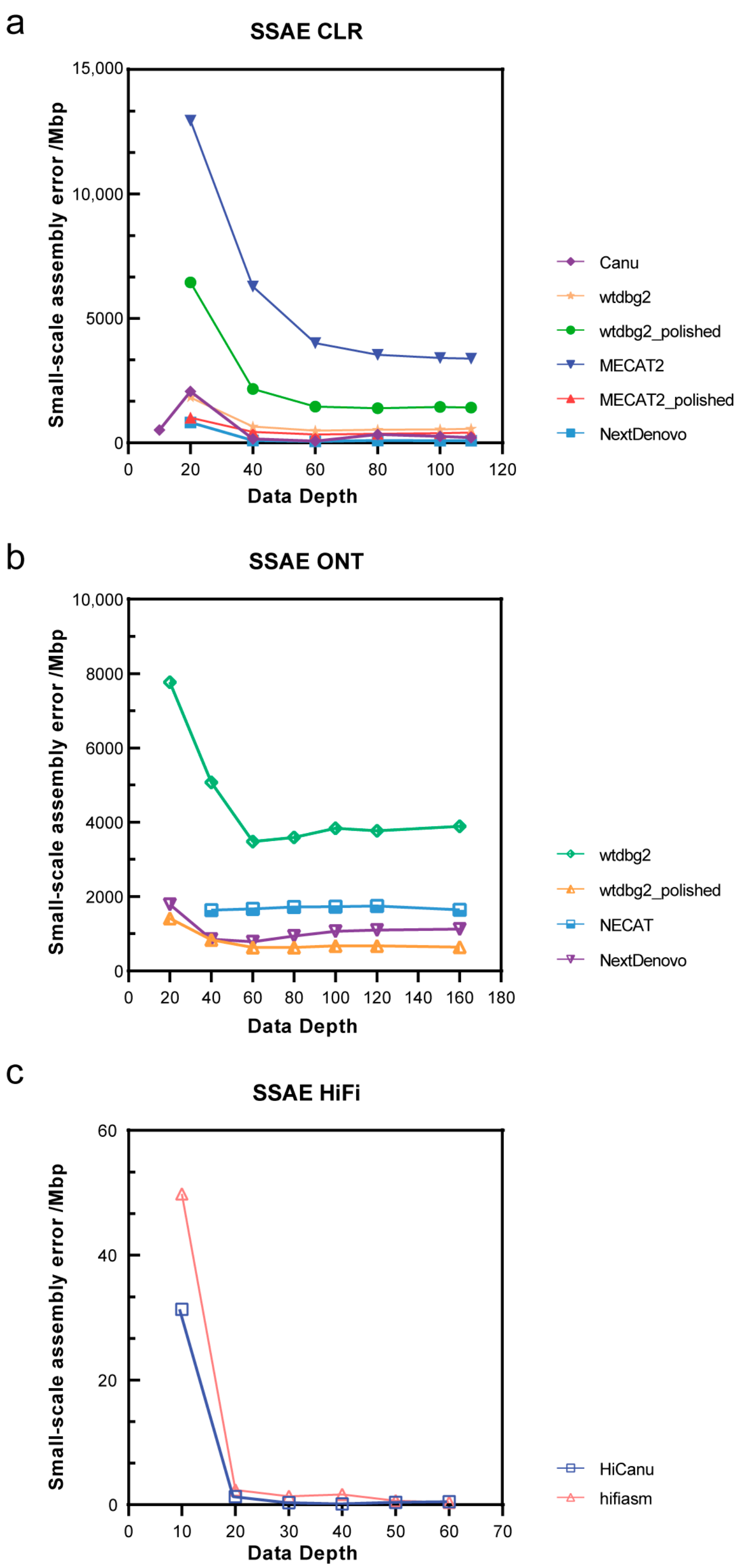

Supplementary Figure S2. Small-scale assembly error (SSAE) of assemblies on (a) CLR, (b) ONT, (c) HIFI subsets with different data depths

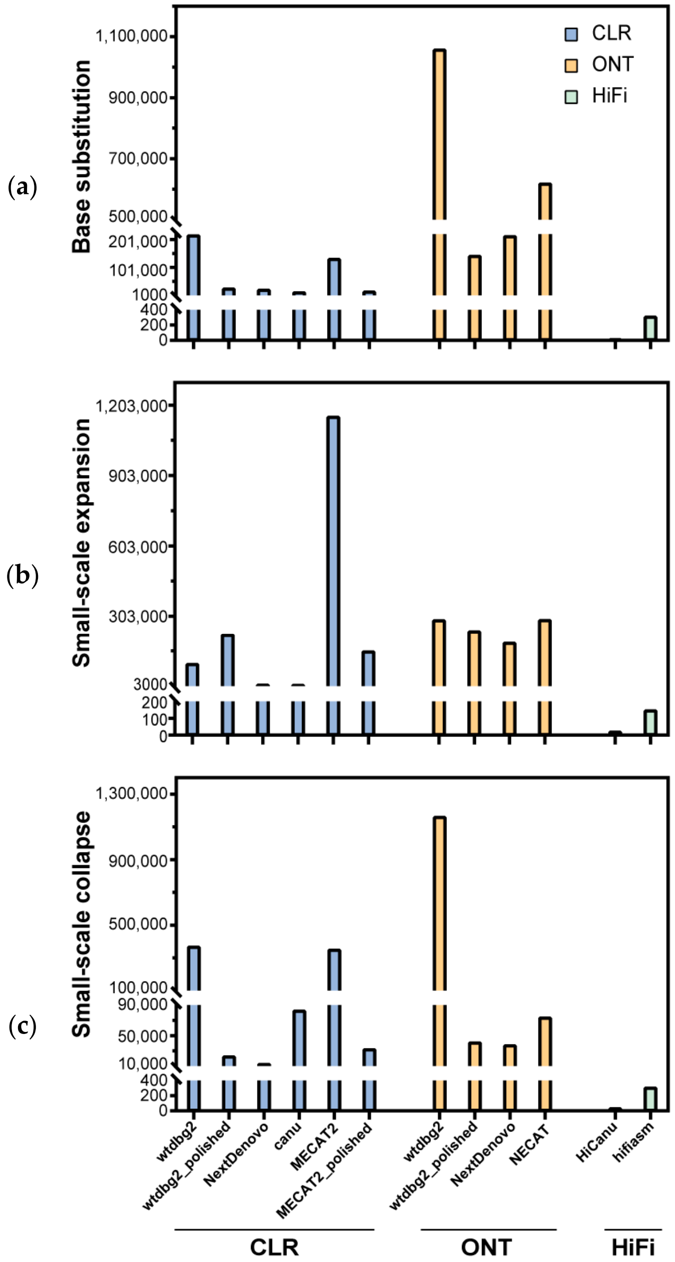

	Base substitution
wtdbg2	216,159
wtdbg2_polished	25,137
NextDenovo	20,658
canu	11,636
MECAT2	131,374
MECAT2_polished	14,099
wtdbg2	1,057,853
wtdbg2_polished	142,558
NextDenovo	213,098
NECAT	618,345
HiCanu	10
hifiasm	309

	Small-scale expansion
wtdbg2	97,140
wtdbg2_polished	222,113
NextDenovo	8644
canu	7534
MECAT2	1,153,660
MECAT2_polished	150,791
wtdbg2	285,891
wtdbg2_polished	237,418
NextDenovo	188,250
NECAT	286,778
HiCanu	20
hifiasm	147

	Small-scale collapse
wtdbg2	367,478
wtdbg2_polished	22,387
NextDenovo	12,758
canu	82,627
MECAT2	349,441
MECAT2_polished	31,926
wtdbg2	1,160,101
wtdbg2_polished	40,850
NextDenovo	37,101
NECAT	73,620
HiCanu	32
hifiasm	305

Supplementary Figure S3. Three types of small-scale assembly error, (a) base substitution, (b) expansion and (c) collapse identified in the best assemblies of CLR (110X), ONT (80X), HIFI (40X) subsets.

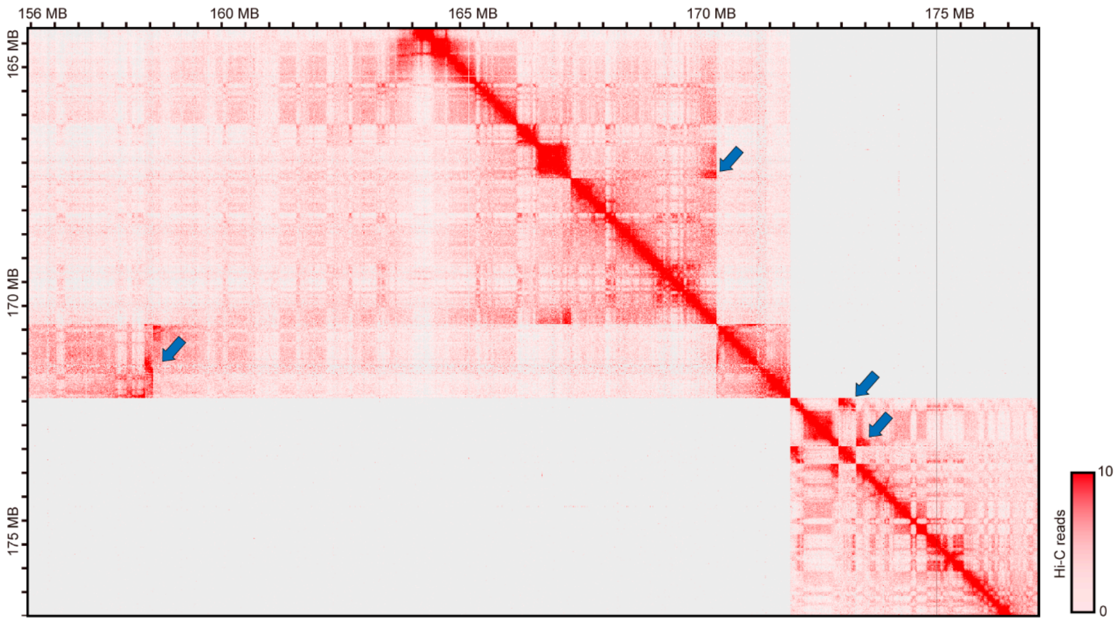

Supplementary Figure S4. Hi-C interaction map of silkworm (D9L) draft assembly. The assembly errors were marked by blue arrows.
